# Interindividual variability in appetitive sensations and relationships between appetitive sensations and energy intake

**DOI:** 10.1038/s41366-023-01436-9

**Published:** 2023-12-22

**Authors:** Eunjin Cheon, Richard D. Mattes

**Affiliations:** Department of Nutrition Science, Purdue University, West Lafayette, IN USA

**Keywords:** Nutrition, Feeding behaviour, Obesity, Obesity, Weight management

## Abstract

**Background:**

Appetitive sensations (AS) are signals that guide eating behaviors. Marked short-term inter-individual variability in AS has been reported but the long-term stability of individual ratings and their dietary implications are not well characterized.

**Objectives:**

This study explored the stability of inter-individual ratings of hunger, fullness and thirst for 17 weeks; determined the relationships between these sensations, eating patterns and energy intake (EI); as well as the associations between ratings and selected individual characteristics (age, gender, BMI).

**Methods:**

A 17-week observational study collected hourly appetitive ratings and dietary intake data from 97 (90 completers, 7 partial completers) healthy adults at weeks 1, 9, and 17.

**Results:**

There were marked and stable inter-individual differences over the 17 weeks for hunger (week 1 vs. week 9, *r* = 0.72 (*p* < 0.001); week 1 vs. week 17, *r* = 0.67 (*p* < 0.001); week 9 vs. week 17, *r* = 0.77 (*p* < 0.001)); fullness (week 1 vs. week 9 *r* = 0.74 (*p* < 0.001); week 1 vs. week 17, *r* = 0.71 (*p* < 0.001); week 9 vs. week 17, *r* = 0.81 (*p* < 0.001)); and thirst (week 1 vs. week 9 *r* = 0.82 (*p* < 0.001); week 1 vs. week 17, *r* = 0.81 (*p* < 0.001); week 9 vs. week 17, *r* = 0.88 (*p* < 0.001)). Cross-correlation functions revealed EI and eating pattern exerted stronger effects on AS than the reverse. However, the absolute effect sizes were small. Path analyses also indicated that there were weak relationships between AS and EI. No robust effects of the studied individual characteristics were observed.

**Conclusion:**

This study found that acute and chronic sensations of hunger, fullness and thirst are relatively stable within individuals but vary markedly between individuals. In addition, the present data indicate AS are poorly associated with dietary patterns or with EI under conditions of relatively stable energy balance.

## Introduction

Weight gain stems from sustained positive energy balance. Appetitive sensations (AS) are viewed as drivers of energy intake (EI) [1, 2] and they oscillate markedly over hours [2]. If they do so in ways that result in energy balance, no change of body weight would be predicted over time. However, if there is a sustained bias towards higher or lower motivation to eat, this may predispose individuals toward positive or negative energy balance and weight change. As both low and high body weight holds health risk, a key question is whether there are reliable inter-individual differences in the mean daily level of AS that may account for differences in longer-term EI and risk of unhealthy weight. Prior shorter-term studies with limited sample sizes suggest there are reliable inter-individual differences in AS [3, 4]. The first aim of this study was to more rigorously explore daily AS ratings over time to determine their stability and the magnitude of inter-individual differences. It was hypothesized that marked and reliable individual differences exist. If true, the next question becomes, what are the implications? Various hypotheses could be proposed to link AS to adiposity status. One holds that individuals with high chronic hunger or low chronic fullness will consume more energy to mitigate these unpleasant sensations and thereby be at increased risk of higher adiposity. Alternatively, high chronic hunger or low fullness may be the result of low EI, a condition likely to be associated with low adiposity. To better characterize these alternatives, the second aim of this work was to explore the directionality of the relationships between AS and eating patterns as well as between AS and EI. Finally, the lack of clarity on the relationship between AS and EI may stem from failure to account for varying contributions by individual characteristics including BMI (body mass index) [5], age [6], and sex [6, 7] in prior work. Each has been hypothesized to modify appetite-diet associations and risk for obesity. However, the literature for each has been mixed: BMI [6, 8, 9], sex [6, 7, 10, 11], age [12–14]. Thus, a third aim was to determine whether these selected individual characteristics consistently map onto patterns of AS intensity over time.

## Methods

### Participants

Participants were recruited through public announcements. Eligibility criteria included (1) healthy without history of chronic diseases, (2) 18–64 years of age, (3) weight stable (body weight fluctuation <2.5 kg over the prior 3 months), (4) not taking medications known to affect appetite, and (5) not planning to change lifestyle behaviors that could affect energy balance. Based on previous studies, 16 to 30 participants are required to detect a 10% difference in appetitive sensations in paired-design trials [15, 16] with 80% power. We set participant recruitment goals based on three age groups (18–30 years, 31–45 years, 45–65 years old) with two BMI categories (lean and overweight/obese) within each age group. Informed consent was obtained from all participants. All procedures involving human subjects were approved by the Purdue University Institutional Review Board and this study was registered in clinicaltrials.gov (NCT04836416).

### Protocol

This was a 17-week observational study. At screening, participants reported their age and sex; completed a battery of questionnaires addressing selected eating traits; and weight and height were measured. In addition, participants received appetite lexicon training as well as general instructions on study activities. After the screening visit (week 0), participation was virtual at study weeks 1, 9, and 17. At each of these timepoints, data were collected on two randomized, non-consecutive weekdays and one weekend day. For each day, participants self-reported their appetite sensations, physical activity and dietary intake. Participants attended a virtual meeting one week before weeks 9 and 17 to remind them of study activities to follow on each of the randomly selected days. The definitions of hunger and fullness were explained again, and any questions were addressed.

### Appetite lexicon training

Participants watched a training video defining four AS (hunger, fullness, desire to eat, and prospective consumption) and a series of video tutorials about common confusions among appetite concepts (see supplemental materials). After they completed the training, participants took an online quiz to confirm their understanding of the concepts. A score of at least 90% correct responses was required to pass the appetite lexicon training. All participants passed the quiz.

### Appetite sensation assessment

Participants rated their perceived hunger, fullness, and thirst on their cell phones/computers via a web based survey every waking hour on three days that were randomly selected at weeks 1, 9, and 17. Two days were non-consecutive weekdays and one day was a weekend day. The questions for appetite sensations (hunger, fullness, and thirst) were “how hungry do you feel”, “how full do you feel?”, and “how thirsty do you feel?”, all were rated from “not at all” anchored at 0% to “extremely” anchored at 100% [15, 17]. Responses were provided on a visual analog scale (VAS). All entries were time and date stamped to ensure the ratings were made at the intended times. Ratings were accepted if they were recorded ± 5 min of each subsequent hour relative to the initial recording. If they either woke up three hours later than their usual time of awakening or missed any three ratings during waking hours, participants were asked to complete the ratings again on another random day.

### Physical activity assessment

Free-living energy expenditure was measured using the ActivityTracker Pedometer mobile app (version 3.4.3.277) the same days that appetite ratings were recorded. The mobile app automatically tracked participants’ steps, calorie usage, active time, and moving distance on their cell phones. Participants were asked to carry their phones throughout these days. They reported their recorded energy expenditure by submitting a screenshot of the mobile app page from their phones the following day through the web-based survey.

### Dietary assessment

Participants reported their 24-h dietary intake at one time on the day after appetite ratings were recorded. Dietary intake was obtained by a dietary recall method using the automated self-administered 24-h dietary recall system (ASA24- version 2020–2022). The system systematically asks participants how many meals they had; what they ate; and how much they ate at each meal. After entering all foods and beverages consumed, the system prompted them to reconsider whether any sauces, condiments or salad dressing as well as foods or beverages may have been omitted. The plausibility of the data was assessed using the Goldberg formula [18].

### Data analysis

#### Aim1: Determine the magnitude and consistency of appetitive sensations (hunger, fullness, and thirst) between individuals

One-way ANOVA was used to investigate within and between individual variances of each AS and to investigate differences in EI, eating frequency, portion size, age, and BMI between AS tertiles. Individuals were divided into three groups based on the total mean of AS over the 17 weeks (9 days = 3 daysX3 weeks). The homogeneity of variances was tested using the Levene’s test. If the variance was not homogeneous, the Welch’s ANOVA *p*-value was used.

A one-way ANOVA was also run to explore seasonal effects on appetitive ratings. Seasons were “Dec–Feb”, “Mar–May”, “June–Aug”, “Sep–Nov”. Mean daily appetite ratings were compared between seasons. The homogeneity of variances were tested using the Levene’s test. If the variance was not homogeneous, the Welch’s ANOVA *p*-value was used.

#### Aim2: Relationships between AS and energy intake and between AS and eating patterns

Pearson’s correlation coefficients were calculated to examine the relationships between daily mean AS ratings; weekly mean appetite ratings (weeks 1, 9, and 17); daily mean AS and EI, portion size, and BMI. Spearman’s correlation coefficients were calculated to investigate the relationship between daily mean AS and eating frequency (eating frequency was coded categorically).

A generalized regression model was used to explore the difference of mean AS between weekdays and weekends since eating patterns or energy intake during weekends and weekends may be different. A linear mixed model was used with the Kenward-Roger approximation for degrees of freedom. Week was set as a random effect and individual was nested within the week. The relationship between the total mean AS of the nine days (3 days X 3 weeks) and EI was determined by a generalized regression model. Age, gender, BMI, and physical activity were included as covariates.

The directionality of relationships between hourly EI and hourly appetite ratings and between hourly drinking events and hourly thirst ratings were determined using cross-correlation function (CCF) analysis. All data from time-series analyses of EI, drinking event, and AS were pre-whitened; a procedure to standardize data for cross-correlation function analyses to reduce bias from autocorrelations [19]. The largest correlation coefficients were used for the interpretation of the possible directionality between appetitive sensations and EI. Mean AS changes one-hour before and after meals was compared using the Wilcoxon signed-rank test (WSR).

The relationships between AS and EI, eating frequency, portion size, and BMI were explored using a path analysis of structural equation modeling (SEM). Data were normalized by min-max scaling before analysis. Model fit was evaluated by chi-square *p*-value, normed fit index (NFI), relative fit index (RFI), incremental fit index (IFI), Turker–Lewis index (TLI), comparative fit index (CFI), and root-mean-squared error of approximation (RMSEA). Good model fit was determined a priori to be a *p*-value of chi-square greater than 0.05, NFI value greater than 0.95, RFI, IFI, TLI, and CFI values greater than 0.9, and RMSEA less than 0.05 [20].

#### Aim3: Individuals characteristics (sex, age, BMI) among AS tertiles

The relationship between AS tertiles and categorical demographic characteristics including sex (male and female), and BMI groups (BMI 18.5–24.9 kg/m^2^(normal), 25–29.9 kg/m^2^ (overweight), 30 kg/m^2^ or greater (obese)) was explored using the contingency test.

Data are expressed as mean ± SE. Statistical significance was determined by α < 0.05, two-tailed for all analyses. Tukey’s adjustments were used as a correction for multiple comparisons. SAS (version 9.4) software was used for correlation, ANOVA, general linear regression, WSR, and contingency testing. IBM SPSS (version 28th) software was used for CCF and IBM SPSS AMOS (version 26) software was used for SEM.

## Results

### Participant demographic characteristics

A total of 1149 participants were screened. Among those, 407 failed to complete the screening; 483 did not meet the inclusion criteria; 47 declined to participate after learning about study activities; and 103 participants were willing to participate but their age group had been filled. Thus, 109 participants were enrolled in the study but 12 dropped in the middle of the first week of the study. Among them, 90 participants completed the study, 7 participants partially completed the study. Attrition was due to time conflicts in 2 cases, but most commonly, no specific reason was provided. Age ranged from 18–64 years with a mean of 33.1 ± 1.2 years (18–30 y/o *n* = 48; 31–49 y/o *n* = 35; 50–64 y/o *n* = 14). The mean BMI was 26.8 ± 0.6 kg/m^2^ (18–24.9 kg/m^2^ *n* = 48; 25–29.9 kg/m^2^ *n* = 28; ≥30 kg/m^2^ *n* = 21). There were 20 males and 77 females.

### Appetite variation within and between individuals

**Fig. 1:**
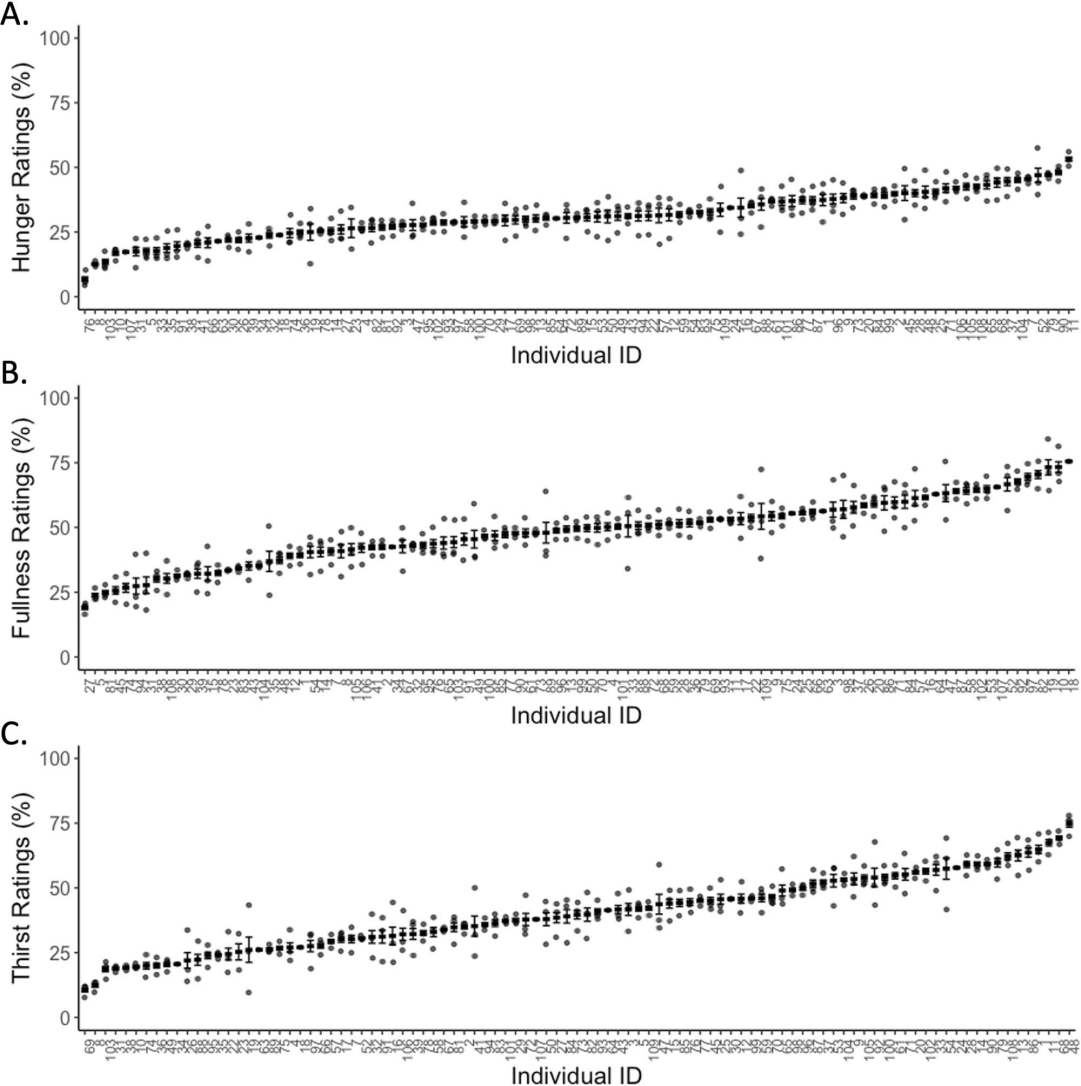
Appetitive sensations of participants in ascending order. **A** hunger ratings, **B** fullness ratings, **C** thirst ratings (• = mean ratings of each week, **−** mean of all weeks, | error bars = standard errors).

There were marked inter-individual differences for each sensation over the 17 weeks: hunger (*p* < 0.0001), fullness (*p* < 0.0001), thirst (*p* < 0.0001) (Fig. 1). Daily mean hunger ratings ranged from 2.9 to 62.5% across individuals. The values for fullness and thirst were 13.4 to 87.7% and 2.5 to 87.6% respectively. Across all appetitive ratings (3 days X 3 weeks), the daily mean variance in ratings between individuals was greater than the mean variance within individuals (hunger: within variance = 52.7 (7.3% of variance), between variance = 670.1 (92.7% of variance), fullness: within variance = 69.0 (5.1% of variance), between variance = 1285.6 (94.9% of variance), thirst: within variance = 59.4 (3.3% of variance), between variance = 1738.9 (96.7% of variance)).

**Fig. 2:**
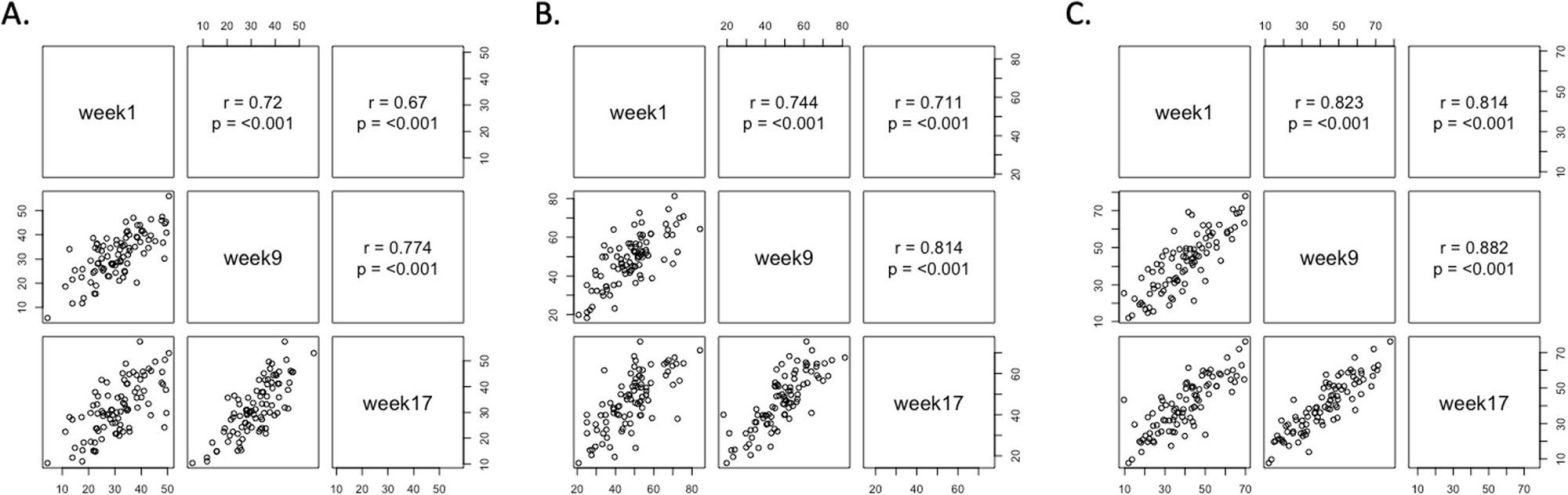
Pearson’s correlations of weekly mean appetitive sensations between weeks. **A** correlations of hunger sensations, **B** correlations of fullness sensations, **C** correlations of thirst sensations.

Pearson’s correlation coefficients were computed for each weekly mean AS as an index of the consistency of AS ratings. Correlations were strong and positive between weeks 1, 9 and 17 indicating AS ratings of individuals were consistent over time (Fig. 2). There was no significant difference in any measured daily mean AS between weekdays vs. and weekend days (one-way ANOVA, hunger: *p* = 0.96, fullness: *p* = 0.92, thirst: *p* = 0.98) (covariate estimate confidence intervals (CI) were: hunger: weekdays (39.9–85.7) vs. weekend (40.4–91.1); fullness: weekdays (75.1–155.9) vs. weekend (92.5–193.1); thirst: weekdays (121.4–230.2) vs. weekend (137.5–265.2)). Appetite ratings in different seasons (“December–February”, “March–May”, “June–August”, and “September–November”) were compared using one-way ANOVA. No significant effect of season of the year on hunger and thirst ratings was observed (one-way ANOVA, hunger: *p* = 0.56, thirst: *p* = 0.54). While there is a significant difference between fullness ratings during “June–August” and ratings during “December–February” (one-way ANOVA, *p* = 0.03), the difference was marginal (Multiple comparisons using Tukey’s adjustment: *p* = 0.02, difference: −4.11%) and no other seasons showed significant differences in fullness ratings. Waking hours ranged from 13 to 17 h (mean = 15 h), thus the total number of hourly ratings differed between participants. However, there was no significant association between number of ratings and hunger, fullness or thirst. The correlation between daily mean hunger ratings and daily mean fullness ratings was weak (*r* = −0.07, *p* = 0.04). There was a moderate correlation between daily mean hunger ratings and daily mean thirst ratings (*r* = 0.47, *p* < 0.001). There was no significant correlation between daily mean fullness and thirst (*r* = 0.02, *p* = 0.6).

The demographic characteristics (including age, sex, and BMI) of those who reported higher and lower AS strengths, were examined by dividing the sample into tertiles. The 1^st^ tertile had the highest mean daily appetitive sensations. For hunger, the mean age in the 3^rd^ tertile (37.8 ± 2.3year-old) was higher than the mean age in the 2^nd^ tertile (29.1 ± 1.7year-old) (*p* = 0.008). No other comparisons of AS based on age, sex or BMI were significantly different.

### Appetitive sensations, energy intake and BMI

**Fig. 3:**
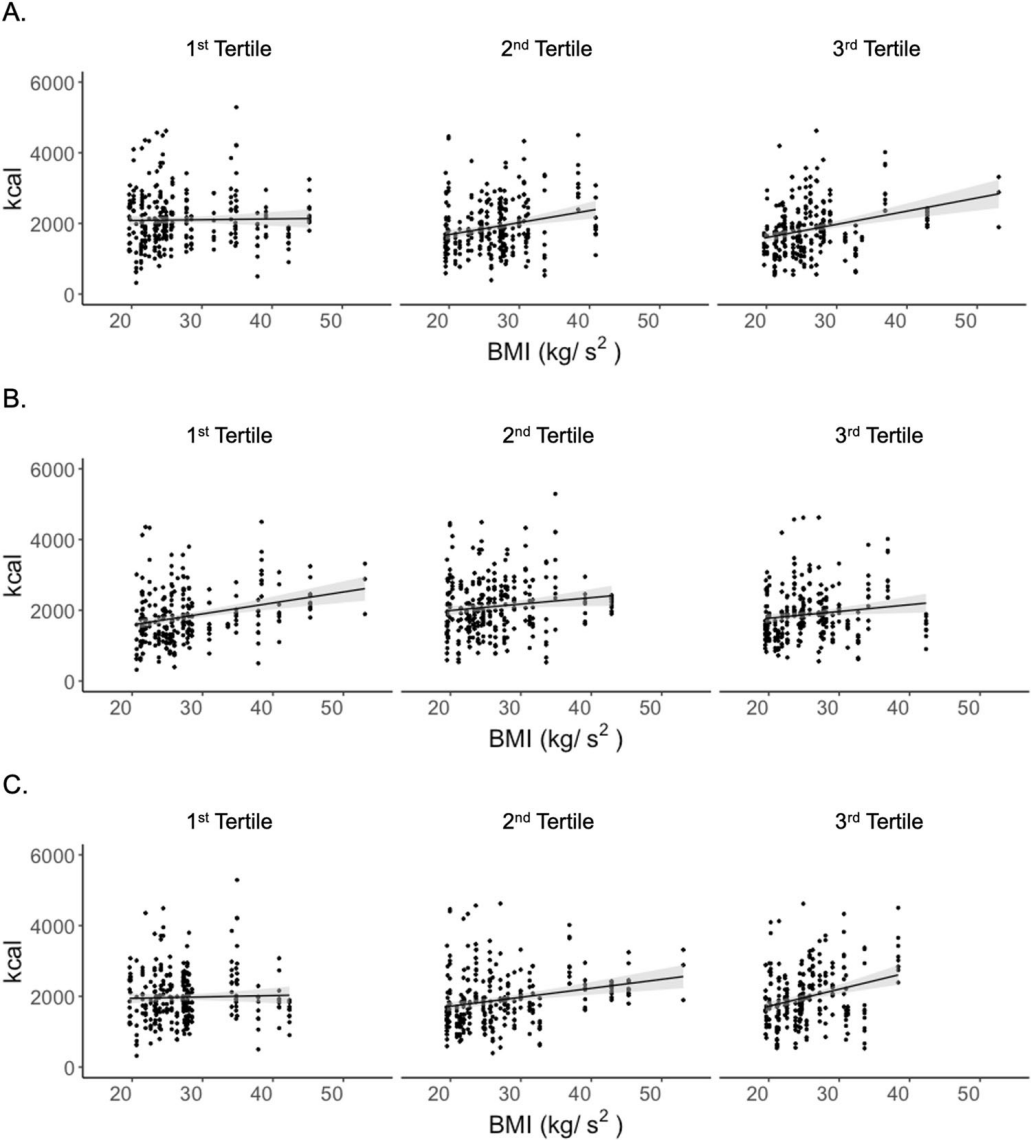
Scatter plots with regression lines between energy intake and BMI of appetitive sensation tertiles. **A** hunger tertiles, **B** fullness tertiles, and **C** thirst ranking tertiles.

The mean EI of the nine test days (3 days X 3 weeks) was not statistically different based on tertiles of hunger (EI of 1^st^, 2^nd^, and 3^rd^ tertiles: 2098 ± 90, 1888 ± 65, 1848 ± 99 kcal), (fullness: 1856 ± 95, 2107 ± 92, 1865 ± 85 kcal) or thirst: (1958 ± 85, 1915 ± 93, 1960 ± 101 kcal). This also held in age, sex, BMI and physical activity adjusted regression models (EI and hunger: *p* = 0.64; fullness: *p* = 0.29; and thirst: *p* = 0.28). EI and BMI were positively associated (*p* < 0.01, *r* = 0.17). Separate regression analyses were conducted between EI and BMI for each AS tertile group (Fig. 3). The slope of 1^st^ tertile participants was lower compared to the slopes of the 2^nd^ and 3^rd^ tertile participants for hunger (slopes of regression lines of 1^st^, 2^nd^, and 3^rd^ tertiles: 2.21, *p* = 0.74; 33.97, *p* < 0.0001; 37.45, *p* < 0.0001) and thirst (3.96, *p* = 0.61; 25.36, *p* < 0.0001; 48.13, *p* < 0.0001). Among 1^st^ tertile groups, no significant associations were observed between BMI and EI for hunger: *p* = 0.74 or thirst: *p* = 0.61. In contrast, a significant association between EI and BMI was observed for fullness in the 1^st^ and 2^nd^ tertile groups, but not the 3^rd^ (slopes of regression lines of 1^st^, 2^nd^, and 3^rd^ tertiles: 30.96, *p* < 0.0001; 18.14, *p* = 0.03; 18.72, *p* = 0.24). The lack of association could be due to true randomness of sensation-response associations or to offsetting bimodal distributions. That is, some individuals with stable high hunger may report this state because of limited energy intake (prediction of a lean phenotype) or because of a strong sensation that is not highly responsive to energy intake (prediction of an obese phenotype). Similarly, individuals with low chronic hunger may report this because of high energy intake (prediction of obese phenotype) or a weak appetitive response (prediction of lean phenotype). Exploration of extremes of the appetitive distributions (e.g., chronic high and low hunger) failed to reveal evidence of such offsetting conditions.

### Directionality of relationships between appetitive sensations and energy intake

**Fig. 4:**
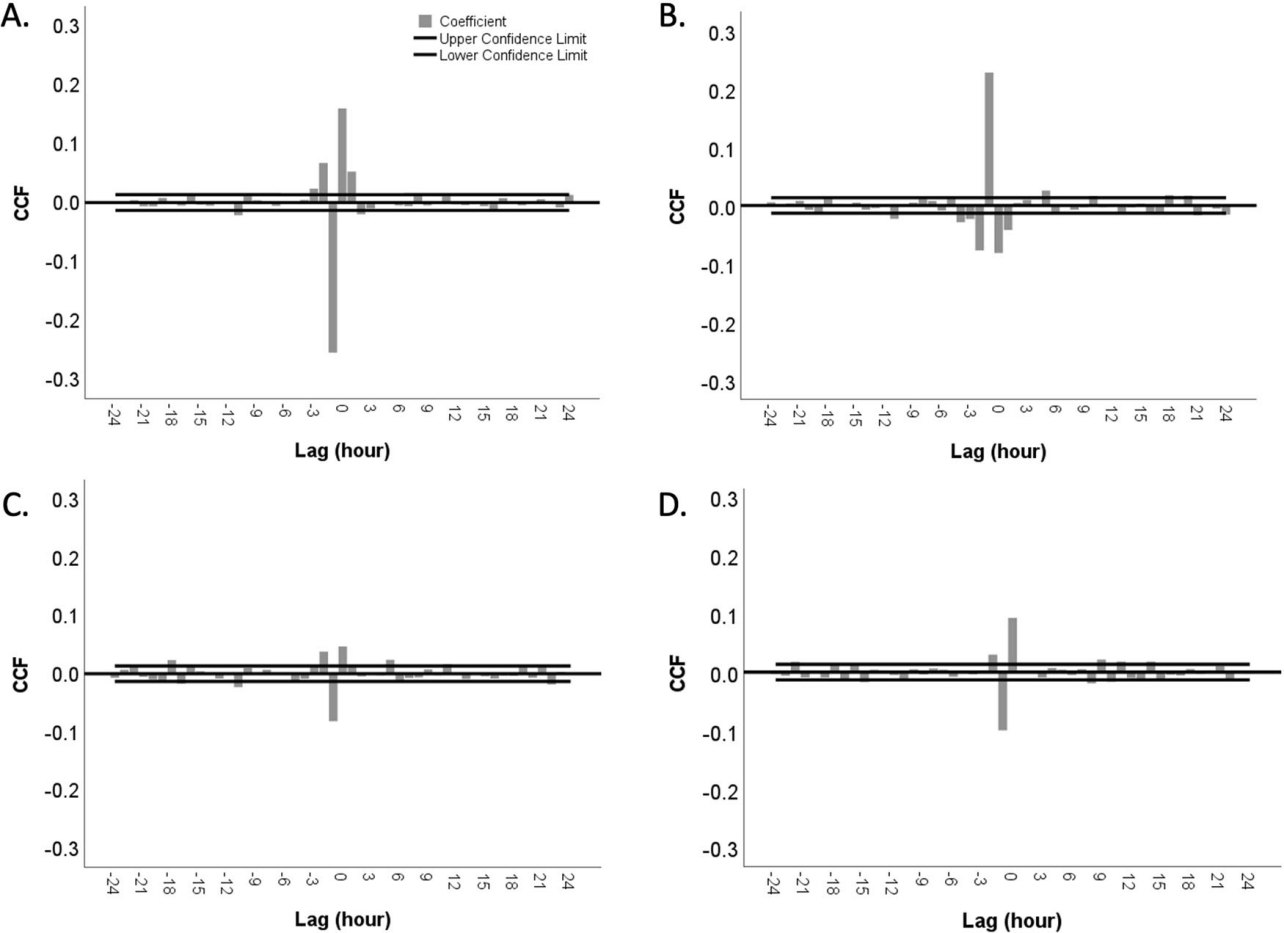
Cross-correlation of appetitive sensations and energy intake with hourly time lags. **A** hunger ratings with energy intake, **B** fullness ratings with energy intake, **C** thirst ratings with energy intake, **D** thirst ratings with drinking event.

To examine the directionality of the relationship between hourly AS and hourly EI, CCF analysis was conducted with a time series of hourly appetite ratings and hourly EI records. Overall, EI leads AS at −1 lag (Fig. 4) meaning EI 1 h earlier was associated with significant changes in AS. The most significant and largest coefficient value of CCF with hunger ratings and EI was −0.255 at −1 lag (Fig. 4A). This reflects the ability of EI to reduce hunger ratings. At +1 lag, there is a significant, but much weaker cross correlation between hunger ratings and EI (coefficient = 0.052, *p* < 0.05) describing how hunger ratings influence EI (Fig. 4A). The largest and statistically significant coefficient value of CCF with fullness ratings and EI is 0.228 at −1 lag indicating EI is associated with increasing fullness ratings (Fig. 4B). Again, there is only a weak cross correlation at +1 lag (coefficient = −0.042, *p* < 0.05) (Fig. 4B). The largest and statistically significant coefficient value of CCF with thirst ratings and EI was only −0.082 at −1 lag, indicating EI is only weakly associated with diminishing thirst ratings (Fig. 4C). CCF with thirst ratings and EI also had a significant, but weak cross correlation at +1 lag (coefficient = 0.013, *p* < 0.05) (Fig. 4C). CCF with thirst ratings and drinking events at −1 lag was significant with a coefficient value −0.1 and it was stronger than the correlation with EI at +1 lag (coefficient = −0.001, *p* > 0.05) (Fig. 4D).

Based on the CCF results, mean changes of AS over the one-hour periods before and after meals were compared. The changes 1-h after meals were greater than the changes 1-h before meals for all sensations: mean changes of hunger–before meals = 4.32%, after meals = −0.96%; fullness–before meals = 2.63%, after meals = 8.06%; thirst–before meals = 3.27%, after meals = −5.52% (WSR, between before and after mean changes of hunger, fullness, and thirst: *p* < 0.0001, *p* < 0.0001, and *p* < 0.0001). This was especially true for the mean changes of hunger ratings (WSR, hunger changes (−14.28%) vs. fullness changes (5.43%), *p* < 0.0001; hunger changes vs. thirst changes (−8.79%), *p* < 0.0001). These results are consistent with the results of the CCF analysis and support the finding that the correlation between EI 1 h earlier and hunger is greater than the correlation between hunger 1 h earlier and EI.

**Fig. 5:**
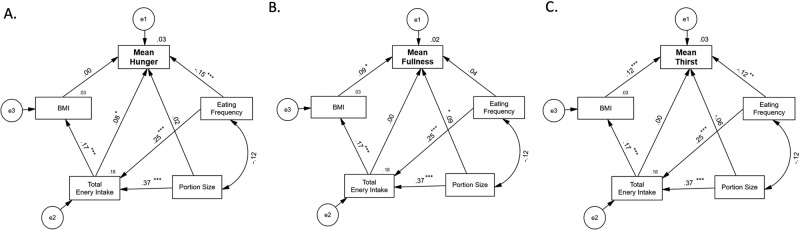
Structural equational models for a path analysis on the relationship between appetitive sensations and energy intake. *p < 0.05, **p < 0.01, ***p < 0.001.

Path analyses, using SEM, were conducted to investigate the effects of daily EI on daily mean AS including the effects of BMI and eating patterns on daily mean AS (Fig. 5). Portion size and eating frequency were include in the model as effects of AS are expected to manifest in changes of EI through one or both of these routes. Physical activity was included in an initial model but, because it exerted no significant effect, it was omitted. When the multiple regression equations were analyzed simultaneously using SEM, there was a significant, but weak, effect of daily EI on daily mean hunger ratings (*p* < 0.001) (Fig. 5A). There was no significant effect of daily EI on daily mean fullness or daily mean thirst ratings (Fig. 5B, C).

### Relationship between eating patterns (eating frequency/portion size) and appetitive sensations

There were significant, but very weak, correlations between AS and eating patterns. Eating frequency was negatively correlated with hunger and thirst ratings (*r* = -0.094, *p* < 0.01; *r* = -0.1, *p* < 0.01, respectively) but no statistically significant relationship was observed with fullness. Portion size was positively correlated with fullness ratings (*r* = 0.092, *p* < 0.01) but no statistically significant relationship was observed for hunger and thirst ratings.

## Discussion

AS incentivize eating decisions. It has been assumed that AS are directly, possibly causally, related to EI so have been the target of considerable study to modulate body weight [1, 2]. Yet, the ability to purposefully control or appropriately respond to AS to achieve desired body weight or BMI goals has proven elusive [21]. This may reflect inadequacies in appetite measurement and/or incomplete understanding of the nature and magnitude of AS contributions to intake. The primary aim of the present work was to gain new insights on longer-term inter-individual variability in AS and its stability over time, as well as the implications of these sensations under naturalistic conditions for eating patterns and EI.

A key assumption in the study of AS is that they are reliable. The present work revealed marked and sustained inter-individual differences in hunger, fullness, and thirst sensations. The stability of AS within individuals was strong across seventeen weeks. This larger trial (*N* = 90) conducted over a longer time period (17 weeks) and with participants representing a range of BMI and age values, confirms reports from several prior studies documenting consistent interindividual differences in AS [3, 22, 23]. One observational trial collected appetite ratings from fifty free-living adults in good health and stable body weight (39 female; 30 ± 11 y/o; BMI 26.3 ± 5.9 kg/m^2^) over one week [22]. They observed a wide distribution of inter-individual mean daily hunger and thirst ratings that were comparable in magnitude to our findings (mean correlation coefficients between days: hunger, *r* = 0.52 ± 0.2; thirst, *r* = 0.78 ± 0.2) [22]. Similar results are reported from intervention trials. One study measured hunger and fullness hourly for 10 h with six different pre-meals in nineteen healthy and weight stable adults (10 males with BMI 22.7–34.5 kg/m^2^, 9 females with BMI 21.2–32.6 kg/m^2^) [23]. The procedure was repeated on two different days. AS changes were not significantly different between meals nor between days [23]. An additional randomized controlled trial involving eighteen healthy men (28.5 ± 9.8 y/o; BMI 27.0 ± 5.0 kg/m^2^) measured postprandial changes of hunger, satisfaction, fullness, and prospective consumption in response to two-repeated standardized meals and two repeated unstandardized meals (*ab libitum* meal) [3]. The AS of participants were consistent between the two repeated measurements regardless of meal types (correlation coefficients between days: hunger, *r* = 0.59; fullness, *r* = 0.41; satisfaction, *r* = 0.74; prospective consumption, *r* = 0.65) [3].

Documentation of large, stable differences in AS naturally raises questions about their dietary and health implications [24, 25]. It is presumed that high hunger drives the initiation of eating and lower fullness facilitates the ingestion of larger portions [24, 25]. The present findings suggest a different interpretation. In the CCF analysis, EI elicited more robust changes of AS than AS changes exerted on EI. Additionally, the mean changes of AS one-hour after an eating event were significantly greater than the mean changes of AS one-hour before an eating event. These findings are also consistent with an earlier cross-sectional study that noted the length of post-meal intervals was consistently positively correlated with subsequent EI (*r* = 0.46, *p* < 0.05) but EI was not significantly correlated with premeal intervals [26]. This association may also hold over longer time intervals as another trial reported prior day EI was correlated with fasting hunger and fullness (*r* = −0.56, *r* = 0.5, respectively) the following day [23]. The present data suggest this directionality also holds for thirst and drinking. Generally, the present data suggests hunger, fullness, and thirst may not be good predictors of EI or drinking. Rather, they may more strongly reflect the consequence of energy or fluid intake. The importance of this asymmetrical relationship has not been examined but may hold useful clinical significance. With respect to energy balance, it has been argued that an eating pattern of multiple smaller eating events, versus fewer larger eating events, will aid weight management [27]. However, data from NHANES [28], the Seventh Day Adventist Trial [29] and others [30–33] indicate this is not the case. Failure to sufficiently reduce the drive to eat after an eating event may result in greater overall daily hunger [34] and thereby enable more unplanned eating events and greater daily energy intake.

Even though an asymmetric relationship between AS and EI has been observed, the association noted between AS and EI was weak in this trial of individuals in relative energy balance. The applicability of these findings to conditions of marked positive or negative energy balance is less clear. It may be hypothesized that at the extremes of energy balance, appetite holds a more important role in eating and weight management. In the larger literature, there is evidence of augmented AS following weight loss [35], but also reports of diminished AS [36] and uncoupling of appetite with intake with weight loss [37]. There are conflicting findings with overfeeding as well, with rapid accommodation of AS to elevated EI [38] and either uncoupling of AS or their ineffectiveness to drive compensation to marked stepwise increments in positive energy balance [39]. This is an area that warrants further study. If AS are weak modulators of EI even under strong energy imbalance conditions, their role in guiding ingestive behavior and body weight to healthful levels is questionable.

There has been and continues to be great interest in the effects of eating frequency and portion size on body weight [27, 28, 32, 40]. In the present trial, there was only a marginal or no significant correlation between AS and either eating frequency or portion size. This finding is consistent with prior work reporting that changes in thirst and hunger 1–3 h prior to a drinking or eating events were only weakly correlated (*r* = −0.06 to −0.09) [4]. Controlled-feeding studies show that eating frequency more than 3X/d reduces peaks of hunger and desire to eat sensations while reduced eating frequency to less than 3 X/d increased these sensations significantly [41]. In contrast, the effect of portion size on AS in RCTs has generally been nonsignificant [42–44]. Other work indicates AS may be affected, but not strongly, by variations in portion sizes of eating events distribution over the day [45]. Taken together, the relationship between AS and eating patterns, defined by eating frequency and portion size, is weak.

Individual characteristics such as BMI, age and sex have been proposed to influence AS, but with varying levels of experimental support. With respect to BMI, multiple studies have compared fasting and/or postprandial appetitive ratings between people who are lean or have obesity and observed no significant group differences [6, 8, 9]. Where positive associations have been noted, they are not robust. For example, a significant correlation with BMI may be noted for one index (e.g., hunger but not fullness) in one study [3]; but a different index in another [46, 47]. Among the tertiles of hunger and fullness sensations in the present study, there was no difference in BMI or BMI category. Because thirst facilitates drinking, and there is evidence individuals with obesity consume higher quantities of energy-yielding beverages [48, 49], we hypothesized that higher thirst would be positively correlated with BMI. However, neither BMI nor BMI category was associated with thirst tertiles in this study. Some previous studies of free-living individuals also reported no associations between BMI and thirst ratings [22] or thirst sensations were even lower in dehydrated individuals with obesity [50].

Some evidence suggests males and females differ in AS [6, 7] but the preponderance of evidence indicates hunger and fullness ratings are not significantly different between men and women in either the fasting [10, 23] or post-prandial [11, 23] state. Thirst was also similar between free-living males and females [22] including under extreme conditions following endurance exercise [51]. The current study also did not find sex differences in AS.

Inconsistent findings have been reported on the effects of age on AS by multiple small trials [12, 13]. Our larger sample of healthy individuals failed to reveal significant differences. A meta-analysis reported that the hunger ratings of older adults (60–88 year-old) were 25 and 39% lower after overnight fasting or in a postprandial state, respectively, compared to younger adults (22–50 year-old) and fullness ratings of older adults were 39 and 37% greater after overnight fasting or in a postprandial state, respectively, than younger adults [14]. However, these findings may reflect deteriorating health [52], medication use [53] and reduced physical activity [54, 55] rather than the aging process. Mixed findings are also reported on the relationship between thirst and fluid intake in older adults [56, 57] that may, again, be related more to medication use and disease burden [58, 59]. Thus, the largely negative findings on associations between AS and BMI, sex and age in this trial are generally in line with the literature. It is not argued that these individual characteristics are unrelated to AS, but rather that they are often superseded by other factors, especially environmental influences [37, 60, 61].

Some limitations of this study warrant mention. First, to reduce respondent burden, we did not measure desire to eat and prospective consumption. These two sensations are also drivers of intake and further research on them will be informative. Second, because this was an observational study, many unmeasured factors might also affect appetite ratings. Third, mis-reporting of energy intakes was prevalent (68 and 20 participants reported lower and higher than expected energy intakes, respectively) as is the case in many trials of free-living individuals [62, 63]. Although this may weaken associations between appetitive sensations and energy intake, the overall directionality of the relationships was supported by multiple analyses including CCF, and correlation.

In summary, this study discovered marked and consistent inter-individual differences in hunger, fullness, and thirst sensations. While the implications for quality-of-life issues were not explored, the associations between AS and dietary patterns and EI were very weak across the range of AS ratings. Stronger associations were observed for the effect of EI on AS than the reverse. If true, this suggests AS hold limited impact on or predictive power for intake. Further, no robust associations between AS and age, BMI, or sex were observed. Whether the relationships between AS and ingestive behaviors differs under more extreme conditions of energy (im)balance warrants further study.

### Supplementary information

Supplemental information

## Data Availability

The data are available upon request.

## References

[1] Hansen TT, Andersen SV, Astrup A, Blundell J, Sjödin A (2019). Is reducing appetite beneficial for body weight management in the context of overweight and obesity? A systematic review and meta‐analysis from clinical trials assessing body weight management after exposure to satiety enhancing and/or hunger reducing products. Obes Rev.

[2] Beaulieu K, Blundell J (2020). The psychobiology of hunger – a scientific perspective. Topoi.

[3] Goltz FR, Thackray AE, Atkinson G, Lolli L, King JA, Dorling JL (2019). True interindividual variability exists in postprandial appetite responses in healthy men but is not moderated by the FTO genotype. J Nutr.

[4] McKiernan F, Hollis JH, McCabe GP, Mattes RD (2009). Thirst-drinking, hunger-eating; tight coupling?. J Am Diet Assoc.

[5] Hopkins M, Blundell JE (2016). Energy balance, body composition, sedentariness and appetite regulation: pathways to obesity. Clin Sci.

[6] Gregersen NT, Møller BK, Raben A, Kristensen ST, Holm L, Flint A, et al. Determinants of appetite ratings: the role of age, gender, BMI, physical activity, smoking habits, and diet/weight concern. Food Nutr Res. 2011;11;55. 10.3402/fnr.v55i0.7028. 10.3402/fnr.v55i0.7028 PMC3160809 21866221

[7] Bédard A, Hudon AM, Drapeau V, Corneau L, Dodin S, Lemieux S (2015). Gender differences in the appetite response to a satiating diet. J Obes.

[8] Clamp L, Hehir APJ, Lambert EV, Beglinger C, Goedecke JH (2015). Lean and obese dietary phenotypes: differences in energy and substrate metabolism and appetite. Br J Nutr.

[9] Mourao DM, Bressan J, Campbell WW, Mattes RD (2007). Effects of food form on appetite and energy intake in lean and obese young adults. Int J Obes.

[10] Leone A, De Amicis R, Pellizzari M, Bertoli S, Ravella S, Battezzati A (2022). Appetite ratings and ghrelin concentrations in young adults after administration of a balanced meal. Does sex matter?. Biol Sex Differ.

[11] Wang GJ, Volkow ND, Telang F, Jayne M, Ma Y, Pradhan K (2009). Evidence of gender differences in the ability to inhibit brain activation elicited by food stimulation. Proc Natl Acad Sci USA.

[12] Parker BA, Sturm K, MacIntosh CG, Feinle C, Horowitz M, Chapman IM (2004). Relation between food intake and visual analogue scale ratings of appetite and other sensations in healthy older and young subjects. Eur J Clin Nutr.

[13] Zandstra EH, Mathey MF, Graaf C, van Staveren WA (2000). Short-term regulation of food intake in children, young adults and the elderly. Eur J Clin Nutr.

[14] Giezenaar C, Chapman I, Luscombe-Marsh N, Feinle-Bisset C, Horowitz M, Soenen S (2016). Ageing is associated with decreases in appetite and energy intake—a meta-analysis in healthy adults. Nutrients.

[15] Flint A, Raben A, Blundell JE, Astrup A (2000). Reproducibility, power and validity of visual analogue scales in assessment of appetite sensations in single test meal studies. Int J Obes.

[16] Jamison RN, Gracely RH, Raymond SA, Levine JG, Marino B, Herrmann TJ (2002). Comparative study of electronic vs. paper VAS ratings: a randomized, crossover trial using healthy volunteers. Pain.

[17] Stubbs RJ, Hughes DA, Johnstone AM, Rowley E, Reid C, Elia M (2000). The use of visual analogue scales to assess motivation to eat in human subjects: a review of their reliability and validity with an evaluation of new hand-held computerized systems for temporal tracking of appetite ratings. Br J Nutr.

[18] Black AE (2000). Critical evaluation of energy intake using the Goldberg cut-off for energy intake:basal metabolic rate. A practical guide to its calculation, use and limitations. Int J Obes Relat Metab Disord.

[19] Probst WN, Stelzenmüller V, Fock HO (2012). Using cross-correlations to assess the relationship between time-lagged pressure and state indicators: an exemplary analysis of North Sea fish population indicators. ICES J Mar Sci.

[20] Beran TN, Violato C (2010). Structural equation modeling in medical research: a primer. BMC Res Notes.

[21] Aukan MI, Nymo S, Haagensli Ollestad K, Akersveen Boyesen G, DeBenedictis JN, Rehfeld JF (2022). Differences in gastrointestinal hormones and appetite ratings among obesity classes. Appetite.

[22] McKiernan F, Houchins JA, Mattes RD (2008). Relationships between human thirst, hunger, drinking, and feeding. Physiol Behav.

[23] Ruddick-Collins LC, Byrne NM, King NA (2019). Assessing the influence of fasted and postprandial states on day-to-day variability of appetite and food preferences. Physiol Behav.

[24] Wardle J, Carnell S (2009). Appetite is a heritable phenotype associated with adiposity. Ann Behav Med.

[25] Wood AC (2018). Gene-Environment interplay in child eating behaviors: what the role of “nature” means for the effects of “nurture.”. Curr Nutr Rep.

[26] Bernstein IL, Zimmerman JC, Czeisler CA, Weitzman ED (1981). Meal patterns in “free-running” humans. Physiol Behav.

[27] Higgins KA, Hudson JL, Hayes AMR, Braun E, Cheon E, Couture SC (2022). Systematic review and meta-analysis on the effect of portion size and ingestive frequency on energy intake and body weight among adults in randomized controlled feeding trials. Adv Nutr.

[28] Zhu Y, Hollis JH (2016). Associations between eating frequency and energy intake, energy density, diet quality and body weight status in adults from the USA. Br J Nutr.

[29] Kahleova H, Lloren JI, Mashchak A, Hill M, Fraser GE (2017). Meal frequency and timing are associated with changes in body mass index in Adventist health study 2. J Nutr.

[30] Bachman JL, Raynor HA (2012). Effects of manipulating eating frequency during a behavioral weight loss intervention: a pilot randomized controlled trial. Obesity.

[31] Cameron JD, Cyr MJ, Doucet É (2010). Increased meal frequency does not promote greater weight loss in subjects who were prescribed an 8-week equi-energetic energy-restricted diet. Br J Nutr.

[32] Kant AK (2014). Evidence for efficacy and effectiveness of changes in eating frequency for body weight management. Adv Nutr.

[33] Palmer MA, Capra S, Baines SK (2009). Association between eating frequency, weight, and health. Nutr Rev.

[34] Ohkawara K, Cornier MA, Kohrt WM, Melanson EL (2013). Effects of increased meal frequency on fat oxidation and perceived hunger. Obesity.

[35] Coutinho SR, With E, Rehfeld JF, Kulseng B, Truby H, Martins C (2018). The impact of rate of weight loss on body composition and compensatory mechanisms during weight reduction: a randomized control trial. Clin Nutr.

[36] Beaulieu K, Casanova N, Oustric P, Turicchi J, Gibbons C, Hopkins M (2020). Matched weight loss through intermittent or continuous energy restriction does not lead to compensatory increases in appetite and eating behavior in a randomized controlled trial in women with overweight and obesity. J Nutr.

[37] Polidori D, Sanghvi A, Seeley R, Hall KD (2016). How strongly does appetite counter weight loss? Quantification of the feedback control of human energy intake. Obesity.

[38] Halliday TM, Rynders CA, Thomas E, Bergouignan A, Pan Z, Kealey EH (2020). Appetite-related responses to overfeeding and longitudinal weight change in obesity-prone and obesity-resistant adults. Obesity.

[39] Jebb SA, Siervo M, Frühbeck G, Goldberg GR, Murgatroyd PR, Prentice AM (2006). Variability of appetite control mechanisms in response to 9 weeks of progressive overfeeding in humans. Int J Obes.

[40] Mattes R (2014). Energy intake and obesity: ingestive frequency outweighs portion size. Physiol Behav.

[41] Leidy HJ, Campbell WW (2011). The effect of eating frequency on appetite control and food intake: brief synopsis of controlled feeding studies. J Nutr.

[42] Kral TV, Roe LS, Rolls BJ (2004). Combined effects of energy density and portion size on energy intake in women. Am J Clin Nutr.

[43] Leidy HJ, Apolzan JW, Mattes RD, Campbell WW (2010). Food form and portion size affect postprandial appetite sensations and hormonal responses in healthy, non-obese, older adults. Obesity.

[44] Rolls BJ, Morris EL, Roe LS (2002). Portion size of food affects energy intake in normal-weight and overweight men and women. Am J Clin Nutr.

[45] Ruddick-Collins LC, Morgan PJ, Fyfe CL, Filipe JAN, Horgan GW, Westerterp KR (2022). Timing of daily calorie loading affects appetite and hunger responses without changes in energy metabolism in healthy subjects with obesity. Cell Metab.

[46] Brennan IM, Luscombe-Marsh ND, Seimon RV, Otto B, Horowitz M, Wishart JM (2012). Effects of fat, protein, and carbohydrate and protein load on appetite, plasma cholecystokinin, peptide YY, and ghrelin, and energy intake in lean and obese men. Am J Physiol Gastrointest Liver Physiol.

[47] Barkeling B, King NA, Näslund E, Blundell JE (2007). Characterization of obese individuals who claim to detect no relationship between their eating pattern and sensations of hunger or fullness. Int J Obes.

[48] Malik VS, Schulze MB, Hu FB (2006). Intake of sugar-sweetened beverages and weight gain: a systematic review. Am J Clin Nutr.

[49] Schulze MB, Manson JE, Ludwig DS, Colditz GA, Stampfer MJ, Willett WC (2004). Sugar-sweetened beverages, weight gain, and incidence of type 2 diabetes in young and middle-aged women. JAMA.

[50] Chang DC, Penesova A, Bunt JC, Stinson EJ, Kavouras SA, Gluck ME (2022). Water intake, thirst, and copeptin responses to two dehydrating stimuli in lean men and men with obesity. Obesity.

[51] Armstrong LE, Johnson EC, McKenzie AL, Ellis LA, Williamson KH (2016). Endurance cyclist fluid intake, hydration status, thirst, and thermal sensations: gender differences. Int J Sport Nutr Exerc Metab.

[52] Mathey MFA. Aging and appetite: social and physiological approaches in the elderly. Wageningen University and Research; 2000.

[53] Pilgrim AL, Robinson SM, Sayer AA, Roberts HC (2015). An overview of appetite decline in older people. Nurs Older People.

[54] Cox NJ, Morrison L, Robinson SM, Roberts HC, Ibrahim K (2021). Older individual’s perceptions of appetite, its loss, influencing factors and adaptions to poor appetite. A qualitative study. Appetite.

[55] de Souto Barreto P (2022). Poor appetite & aging: the role of physical activity under a geroscience perspective. J Nutr Health Aging.

[56] Bossingham MJ, Carnell NS, Campbell WW (2005). Water balance, hydration status, and fat-free mass hydration in younger and older adults. Am J Clin Nutr.

[57] Stookey JD (2005). High prevalence of plasma hypertonicity among community-dwelling older adults: results from NHANES III. J Am Diet Assoc.

[58] Kenney WL, Chiu P (2001). Influence of age on thirst and fluid intake. Med Sci Sports Exerc.

[59] Millard-Stafford M, Wendland DM, O’Dea NK, Norman TL (2012). Thirst and hydration status in everyday life. Nutr Rev.

[60] Cruwys T, Bevelander KE, Hermans RCJ (2015). Social modeling of eating: a review of when and why social influence affects food intake and choice. Appetite.

[61] Zheng H, Lenard NR, Shin AC, Berthoud HR (2009). Appetite control and energy balance regulation in the modern world: reward-driven brain overrides repletion signals. Int J Obes.

[62] Hill RJ, Davies PS (2001). The validity of self-reported energy intake as determined using the doubly labelled water technique. Br J Nutr.

[63] Kim EK, Fenyi JO, Kim JH, Kim MH, Yean SE, Park KW (2022). Comparison of total energy intakes estimated by 24-hour diet recall with total energy expenditure measured by the doubly labeled water method in adults. Nutr Res Pract.

