# Supplementary material for: Interindividual variability in appetitive sensations and relationships between appetitive sensations and energy intake
Source: Int J Obes (Lond). 2023 Dec 22;48(4):477–85. doi: 10.1038/s41366-023-01436-9 (PMC10978491; doi:10.1038/s41366-023-01436-9)
Supplement: Supplementary file 1 — Supplemental information [file 41366_2023_1436_MOESM1_ESM.docx]

Supplemental Data 1

1. Detail procedure of anthropometrics

Height and weight were measured once at the screening meeting. Participants were asked to remove shoes and socks and heavy jackets or coats and to empty their pockets. A medical wall-mounted stadiometer (Seca, Chino, CA) was used to measure height, and a Tanita Body Composition Analyzer (Model TBF-410GS, Tanita Inc., Arlington Heights, IL) was used to measure weight to permit calculation of BMI. For those who were not able to visit the laboratory (e.g., due to geographical distance), height and weight were measured during a virtual meeting through Zoom. Participants were asked to join the meeting with a third party who was able to measure their weight and height at the beginning of the meeting. Before measuring weight and height, a participant was asked to remove shoes and socks and heavy jackets or coats and to empty their pockets. The participant stood straight against the wall, and the third party measured height from the floor to the highest point of the head using an augmented reality technology-based app (either one of the following: Measure app by Apple, AR Ruler App by Google Play, Quick Measure by Samsung). Weight was measured on a scale available to each participant. Participants stood on the scale, and a third party took a picture of the weight measurement when the number had not changed for three seconds. Both height and weight measurements were repeated three-times and the average of three values was used for the study estimate. All the measurements were consistent within ± 1 inch for height and ± 0.5 lbs for weight. A photo of the weight measurement and a screenshot of the height measurement were submitted through Qualtrics.

2. Appetite lexicon training

The appetite lexicon training focused on four major appetitive sensations (hunger, fullness, desire to eat, prospective consumption) as well as thirst. Participants first learned the definition of each appetitive sensation. The definitions were: Hunger is a sensation that motivates the initiation of an eating event and it stems from a biological need for energy. Desire to eat is also a sensation that motivates the initiation of an eating event but stems more from cognitive and sensory cues. Thirst is a sensation that motivates a drinking event. Fullness is a sensation that terminates an eating event. Prospective consumption is an anticipated portion that may be consumed. Common confusions between the concepts were then highlighted. To improve learning efficiency, a learning-by-mistake strategy was used where participants answered questions with specific situations related to common confusions between two different appetitive sensations and then received clarification of the differences between them. There were six sets of questions and explanations about the confusions (hunger vs fullness, hunger vs desire to eat, hunger vs prospective consumption, fullness vs prospective consumption, fullness vs desire to eat, desire to eat vs prospective consumption). This was followed by a quiz and participants were required to score at least 90% correct to remain in the study. All did so.
